# Gaps in maternal-fetal interface rejection response: chronic histiocytic intervillositis

**DOI:** 10.3389/fimmu.2025.1625701

**Published:** 2025-09-19

**Authors:** Xiaoqian Zhang, Zhenya Fang, Xietong Wang

**Affiliations:** ^1^ Key Laboratory of Maternal & Fetal Medicine of National Health Commission of China, Shandong Provincial Maternal and Child Health Care Hospital Affiliated to Qingdao University, Jinan, China; ^2^ Department of Obstetrics and Gynaecology, Shandong Provincial Hospital Affiliated to Shandong First Medical University, Jinan, China; ^3^ School of Clinical and Basic Medicine (Institute of Basic Medicine), Shandong First Medical University (Shandong Academy of Medical Sciences), Jinan, China

**Keywords:** CHI, placental inflammation, macrophage, maternal-fetal interface, alloantigen

## Abstract

Chronic Histiocytic Intervillositis (CHI) is a severe placental inflammatory response caused by various atypical antigens, attracting attention due to its high recurrence rate, which results in adverse pregnancy outcomes such as miscarriage and fetal growth restriction. The pathogenesis of CHI is still poorly understood. Immune factors such as autoimmune diseases or viral infections, maternal-fetal genetic compatibility, and other factors cause immune imbalance at the maternal-fetal interface. Disorders of immune tolerance in CHI includes abnormal activity of *Cytotrophoblasts*, *mononuclear macrophages*, and *CD8^+^*/*CD4^+^ T lymphocytes*. Additionally, pro-inflammatory factors such as IL-1β, TNF-α, and anti-inflammatory molecules like IL-10, TGF-β, and fibrin are crucial in regulating the pathological formation of CHI. Histopathological sections and staining, serological screening, and medical imaging techniques are the primary methods for diagnosing CHI. Patients with CHI may benefit from treatments including immunosuppressants, anticoagulants, and monoclonal antibodies.

## 1 Introduction

Chronic histiocytic villous interstitial inflammation (CHI), also known as chronic villous interstitial inflammation of unknown etiology (CIUE), is a severe inflammatory disease that significantly affects the villous spaces (1–3). CHI distinguishes from chronic villitis, chronic deciduitis, or chronic chorionic amnionitis, although CHI may coexist with chronic villitis in the same inflamed placenta (4–9) (**Table 1**). CHI patients often exhibit severe adverse pregnancy outcomes and a high recurrence rate, associated with complications such as miscarriage and fetal growth restriction (10). However, the understanding of the pathogenesis, diagnosis, and treatment strategies for CHI remains in its infancy.

**Table 1:** Comparison of chronic inflammation of the placenta.

Disease name	VUE (6, 7)	Chronic chorioamnionitis (3, 8)	Chronic deciduitis (9)	CHI (4, 5)
Location of inflammation	Subchorionic or placental parenchyma	Chorionic trophoblast or chorioamnion	Decidua Basalis	intervillous space
Incidence	5%-15%	2%-8%	premature infant 8%-25% term placenta2%-13%	0.06%
Recurrence Rate	10%-15% (High-grade VUE)	–	–	67%-100%
Inflammatory Cells	Maternal T lymphocytes, monocytes	CD3^+^ or CD8^+^ T lymphocytes	Maternal lymphocytes, plasma cells, macrophages	CD68^+^ cells, CD4^+^ T, CD8^+^ T
Clinical features	IUGR and fetal death	Premature birth	Premature birth, abortion	Preeclampsia, abortion, IUGR
Pathological Features	Villous parenchyma lymphocytic infiltration, Villous fibrosis or necrosis	Lymphocytic infiltration of smooth chorion, Patchy trophoblastic necrosis	Lymphocytic infiltration of smooth chorion. Patchy Trophoblastic necrosis	Villous interval monocytic aggregation. Fibrin deposition

VUE, villitis of unknown etiology; CHI, chronic histiocytic intervillositis; IUGR, intrauterine growth restriction.

Although maternal-fetal interface immune tolerance imbalance plays a crucial role in the pathogenesis of CHI, the mechanisms by which maternal-fetal genetic compatibility or immune factors regulate the etiology of CHI, as well as the underlying pathophysiological processes, remain poorly understood. This paper reviews the epidemiological characteristics of CHI, summarizes the biological mechanisms underlying its etiology and pathogenesis, and outlines current diagnostic and clinical treatment approaches, aiming to provide new insights into the regulation of maternal-fetal interface immunity and explore novel strategies for the treatment of placental inflammation.

## 2 CHI is characterized by adverse pregnancy outcome and high recurrence

The prevalence of recurrence for CHI can range from 25% to 100%, displaying significant heterogeneity (10). A study involving 24 patients found that the recurrence rate of adverse outcomes in CHI patients reached 67% (11). Another multicenter prospective study indicated that the recurrence rate of adverse pregnancy outcomes in CHI patients could be as high as 30%. Additionally, patients with a history of severe placental lesions face an even higher risk of CHI recurrence (12).

The following example uses severe adverse pregnancy outcomes such as fetal growth restriction and miscarriage to illustrate the relationship between CHI and severe adverse pregnancy outcomes, as well as their epidemiological characteristics.

### 2.1 CHI and fetal growth restriction

Patients with CHI exhibit significant pathological changes in placental function, and multiple studies have confirmed its association with fetal growth restriction (FGR). Research indicates that the incidence of fetal growth restriction among CHI patients ranges from 51.6% to 73%. Clinical observations of 111 CHI patients revealed that the rate of FGR was significantly higher than that of the control group (70.4% vs. 0.9%, p < 0.001) (13). An analysis of 69 CHI cases from 1977 to 2009 revealed an FGR incidence of 66.7%, with a live birth rate of less than 54%. Similarly, a Dutch cohort study found that among 38 CHI patients, the incidence of FGR was 51.6%. These cases were also associated with microstructural abnormalities, including placental vascular lesions and fibrosis in the chorionic mesenchyme.

### 2.2 CHI and miscarriage

CHI is correlated with fetal miscarriage. Statistics indicate that the rate of spontaneous miscarriage among CHI patients ranges from 9.5% to 33%, with early miscarriages occurring more frequently than late ones. A pathological analysis of 178 placentas in France revealed that 73% of CHI cases were associated with fetal growth restriction, with 9.5% resulting in miscarriage (5). A study involving 69 pregnancies with CHI found that the rate of early spontaneous miscarriage was 30.4%, while the rate for late miscarriage was 13.0% (14). An analysis of 151 CHI cases in France from 2000 to 2020 indicated that early miscarriages constituted 20.0%, while late miscarriages accounted for 4.8% (5). Additionally, the occurrence of CHI is often associated with recurrent miscarriages. In a study conducted in Leiden, Netherlands, the miscarriage rate among 38 women with CHI reached as high as 42% (15).

## 3 The pathophysiological mechanism of CHI

The fetus, as a semi-allogeneic transplant, inducing the mother to form specific tolerance due to multiple synergistic mechanisms. First, trophoblast cells selectively express non-classical HLA molecules (such as HLA-G, HLA-C) to avoid recognition and attack by the mother’s T cells and NK cells, and together with the decidua form a physical barrier (16, 17). Second, local immune cells are reprogrammed. Induced by the high expression of inhibitory HLA-G/E/F receptors on trophoblast cells, regulatory T cells (Treg) and M2 macrophages proliferate, while uterine NK cells and tolerogenic dendritic cells secrete IL-10 and TGF-β, suppressing Th1/Th17 inflammatory responses and maintaining Th2 dominance (18, 19). Concurrently, cytokines promote and maintain an immune tolerance environment. Indoleamine 2,3-dioxygenase depletes tryptophan, while immune checkpoint molecules such as PD-L1/CTLA-4 block T cell activation (20). Trophoblast exosomes carrying miRNAs train maternal immune cells; additionally, the embryo actively “educates” maternal immunity by secreting TSLP and IL-35 (21).

Maternal immune dysfunction is the primary factor breakdown of maternal–fetal immune tolerance and trigger CHI. Studies have shown that patients with autoimmune diseases have an increased risk of CHI. Patients with autoimmune diseases exhibit significant maternal-fetal immune dysfunction and disruption of immune tolerance. Among CHI patients, 58.3% (7/12 cases) had concomitant autoimmune diseases such as systemic lupus erythematosus or antiphospholipid antibodies. A German research group reported in 2023 that 29% (7/24) of 24 CHI patients had autoimmune diseases, and those with positive autoantibodies had significantly increased placental inflammation (p<0.05) (12). Pathogen infections during pregnancy can also trigger similar CHI symptoms (22). Pathogen infections can cause maternal immune dysfunction and maternal-fetal interface homeostasis imbalance during pregnancy. SARS-CoV-2 placental infection during pregnancy can lead to pathological changes in the villous spaces, manifested as chronic histiocytic villous interstitial inflammation combined with trophoblast necrosis (23). Pathological analysis of infection-related CHI revealed that viral particles were detectable in 18.2% of Hofbauer cells and 9.1% of villous capillary endothelial cells, accompanied by acute placental dysfunction and fetal hypoxia (24). Cytomegalovirus can also cause villous inflammatory pathological changes similar to CHI (25).

However, in transplant rejection reactions, MHC molecule recognition plays a central role, so poor maternal-fetal MHC compatibility is a potential triggering factor for CHI. Studies indicate that 30–40% of pregnant women can be detected to express antibodies against paternal HLA during pregnancy, with the proportion increasing with the number of deliveries (26). CHI has been confirmed to meet the Banff antibody-mediated rejection criteria (defined by diffuse infiltration of monocytes) (27). Additionally, twin pregnancy studies have confirmed that genetic factors play a significant role in the development of CHI. In monozygotic twins, CHI occurred bilaterally in the placenta (3/5 cases, with identical genetic backgrounds in both placentas (8), while in dizygotic twins, CHI incidence was inconsistent between the two placentas (3/3 cases, with non-identical genetic backgrounds in both placentas) (28).

However, unlike rejection reactions caused by organ transplantation, the semi-allogeneic transplantation rejection reaction at the maternal-fetal interface is centered on monocytes, with T lymphocytes playing a primary role.

## 4 CHI cytopathic alterations

The cellular pathological mechanism of CHI is primarily characterized by an imbalance proliferation and migration of immune cells at maternal fetal interface (29). Under normal circumstances, the mother maintains immunological tolerance for the fetus through special immune system, such as *Treg*-mediated immunosuppression and *decidual natural killer cells* (*dNK cells*). However, in CHI, this maternal-fetal interface becomes unbalanced (30). Changes in the maternal-fetal interface and immune disorders and alterations in CHI include large number of *CD68^+^ monocyte-macrophages* are abnormally aggregated in the intervillous space, while *T cells* subsets predominantly composed of *CD4^+^*/*CD8^+^ T lymphocytes* significantly infiltrate this space, *Treg cells* reduction, secretion of immune factors such as IL-1β and TGFβR1, and release of immune effector molecules such as MMPs.

### 4.1 Trophoblast

The s*yncytiotrophoblast* (*STB*) develops from the fusion of trophoblast cells in the blastocyst’s outer layer, serving as the core functional unit of the maternal-fetal interface. It is central to nutrient and material exchange in the placenta, secreting hormones like human chorionic gonadotropin (hCG) and progesterone to maintain placental development and function. Importantly, the *STB* also plays a crucial role in immune tolerance and regulation at the maternal-fetal interface, In the placentas of CHI patients, STB was observed in the inflammatory lesion region (31). Previous studies found that normal *syncytiotrophoblasts* do not express MHC class II molecules. However, recent research has shown that MHC class II molecules could be detected on *syncytiotrophoblasts* layer in a pathological condition, which may underlie immune recognition and inflammatory responses in CHI (32).

CD200 is a type I membrane glycoprotein widely expressed in fetal-derived placental trophoblast cells (33). At the maternal-fetal interface, the high expression of CD200 on fetal membrane glycoproteins binds to CD200R on the surface of maternal myeloid immune cells, inhibiting the NF-κB/MAPK pathway through phosphatase cascade. Thereby, the signals break the maternal immune system (34). It inhibits dendritic cell maturation and antigen presentation, drives macrophages toward anti-inflammatory M2 polarization (35). simultaneously downregulates Th1-type pro-inflammatory factors (IFN-γ, TNF-α), and upregulates Th2-type anti-inflammatory factors IL-10, thereby inducing Treg expansion and Th2 shift (33). The CD200/CD200R axis also synergizes with molecules such as indoleamine 2,3-dioxygenase (IDO) and FasL to form a fetal “invisibility cloak,” preventing maternal rejection (36). The expression level of the anti-inflammatory molecule CD200R in CHI placental villous trophoblast cells is significantly reduced, which may be associated with abnormal Treg proliferation and immune homeostasis imbalance at the maternal-fetal interface (37).

Besides, the exonuclease CD39 is an immune signaling molecule highly expressed on the surface of trophoblast cells. CD39 hydrolyzes extracellular pro-inflammatory ATP/ADP to generate AMP, which is further converted by CD73 into the immunosuppressive adenosine, thereby establishing a “high adenosine-low ATP” microenvironment at the maternal-fetal interface (38). Adenosine is the core molecule in the formation of an inhibitory immune homeostasis. Adenosine binds to the A2A receptor on maternal immune cells, inhibiting NK cell cytotoxicity, T cell activation, and DC maturation, while promoting Treg expansion and M2 macrophage polarization (39, 40). It also synergizes with tolerance molecules such as PD-L1 and HLA-G to prevent maternal rejection of the semi-allogeneic fetus (41). The reduced expression of the immunosuppressive enzyme CD39 in the trophoblast cells of the placenta disrupts the immune tolerance at the maternal-fetal interface (42).

Additionally, the elevated expression of the adhesion molecule ICAM-1 in the trophoblast cells enhances the adhesion capacity of macrophages, facilitating their accumulation in the intervillous space (43). Furthermore, the increased expression of the inflammatory receptor TLR1 in the trophoblast layer promotes the formation of inflammation in the intervillous space (44).

In CHI placental lesions, trophoblast cells induce large numbers of CD68^+^ monocytes in the villous spaces, which is also the primary pathological feature of CHI.

### 4.2 Macrophages

Macrophages are one of the primary immune cells at the maternal-fetal interface. Research indicates that in the CHI placenta, macrophages primarily originate from maternal circulation rather than from *fetal Hofbauer cells*. The infiltration of macrophages predominates in this lesion, accounting for 80% of inflammatory cells (45). *Macrophages* play crucial roles in antigen presentation, immune modulation, and the repair of inflammatory damage. In the CHI lesion, macrophages display a mixed *M1*/*M2* polarization phenotype. The *M1* subset typically expresses high levels of pro-inflammatory factors such as Tumor Necrosis Factor-alpha (TNF-α) and Interleukin-6 (IL-6), while the *M2* subset suppresses local T cell activation by secreting Interleukin-10 (IL-10) and Transforming Growth Factor-beta (TGF-β) (46). Furthermore, studies on CHI have found that macrophages synthesize and release Interleukin-1 beta (IL-1β) through the NLR family pyrin domain containing 3 (NLRP3) inflammasome pathway, leading to its maturation and release, which in turn induces placental inflammation (47). It is important to note that macrophages mediate the remodeling of the extracellular matrix and tissue repair during placental inflammation in preeclampsia (48), and they are also a significant source of proteins for collagen deposition and fibrosis fetal interface in parturition and preterm birth (49).

Macrophages are the primary cells involved in immune regulation and antigen presentation. In classical immune responses, macrophages present antigens bound to MHC class II molecules to *CD4^+^ T* lymphocytes, which constitutes a critical step in the immune response.

### 4.3 T lymphocytes

As the most numerous immune cells in the body, *T lymphocytes* play a central role in immune recognition and response at the maternal-fetal interface (50). *CD4^+^ T cells* and *CD8^+^ T cells* are the effector cells responsible for self-limiting immune recognition of antigens, which trigger immune rejection responses. In CHI, there is a significant presence of *CD4^+^ T cells* and *CD8^+^ T cells* in the placental villous space, with their ratio being nearly 1:1.

A plethora of research has indicated that the expression of *cytotoxic T lymphocytes* (*CTL*), a population of immune cells differentiated from *CD8^+^ T cells*, is elevated in patients diagnosed with Chronic Hypertension in Pregnancy in comparison to levels observed during a normal pregnancy (51). During the immune induction phase of inflammation in CHI, *CD8^+^ T cells* may recognize paternal antigen-MHC I complexes presented by macrophages, which is similar to the classical immune recognition pathway. This recognition leads to their activation, proliferation, and differentiation into *CTL cells*, exerting cytotoxic effects. *CTL cells* could induce target cell apoptosis by expressing perforin and granzyme B through the Fas/FasL pathway.

Besides, *CD4^+^ T cells* play the core role in immune transplant rejection. *CD4^+^ T cells* are activated by recognizing MHC-II molecule-antigen complex and differentiates into Th1 lymphocytes, which assist *CD8^+^ T cells* in exerting cytotoxic functions. The expression of MHC II-antigen complex by trophoblasts in CHI is a potential activation binding site for *CD4^+^ T cells*. *CD4^+^ T cells* may play a role in CHI similar to that in liver transplant rejection, where IFN-γ and TNF-α secreted by Th1 cells exacerbate the inflammatory response, disrupt immune tolerance, and trigger a cascade amplification of the inflammatory response (52).

Additionally, Treg cells are vital for the development and maintenance of placental function. The appropriate number and functionality of Tregs are critical for achieving maternal-fetal immune tolerance. Insufficient Treg numbers and activity can lead to complications such as recurrent miscarriage and preeclampsia (53). Tregs bind to CD80 and CD86 on *antigen-presenting cells* (*APCs*) through cytotoxic T lymphocyte-associated protein 4 (CTLA-4), competitively inhibiting costimulatory signals for *CD4^+^T cells* (54). A significant infiltration of *Tregs* is observed in the CHI chorionic space, accompanied by reduced Foxp3 expression. This situation reflects a compensatory mechanism for the imbalance at the maternal-fetal interface and immune tolerance in CHI (55). Additionally, Treg cells secrete immunomodulatory factors like TGF-β to suppress the activity of *CD4^+^ T cells*, preventing maternal immune rejection of the fetus (56).

CHI placental pathology is multi-layered and multi-faceted, involving not only cellular changes but also significant molecular alterations.

## 5 Cytokines in CHI placental inflammation

The core pathological mechanism of CHI involves a dynamic imbalance in the cytokine network. These cytokines regulate the inflammatory process through a complex interactive network. They can be categorized into pro-inflammatory and anti-inflammatory cytokines based on their functional characteristics. Pro-inflammatory cytokines (such as IL-1, IL-6, and TNF-α) serve as the primary drivers of the inflammatory cascade, facilitating the recruitment and activation of immune cells. Anti-inflammatory cytokines (such as IL-10 and TGF-β) help suppress excessive immune responses and maintain immune tolerance and homeostasis.

### 5.1 IL-1β

IL-1β plays a crucial role in placental inflammation and is implicated in various pregnancy complications, particularly in placental immune regulation. It is likely that IL-1β is secreted by infiltrating macrophages and trophoblast cells via the NLRP3 inflammasome pathway. IL-1β promotes trophoblast invasion and angiogenesis by activating the PI3K/Akt-VEGF pathway. It also enhances maternal-fetal immune tolerance by inducing NK cells in the decidua to secrete IL-8 and GM-CSF (57). In addition, low concentrations of IL-1β upregulate HLA-G expression in trophoblast cells while simultaneously suppressing the excessive activation of maternal T cells (58).

In the placenta of CHI, the expression level of the IL-1β gene increases by 3.9 times, according to reference (59). Under pathological conditions (such as preeclampsia and chronic villitis), macrophages trigger the secretion and synthesis of sFLT-1 through a pro-inflammatory cascade involving NLRP3 inflammasomes and Gasdermin D (GSDMD) (60). These proteins antagonize the binding of VEGF-A and PlGF to the vascular endothelial cell receptor VEGFR-1/2, leading to increased NO synthesis, elevated ROS production, and heightened vascular permeability, thereby inducing vascular spasm (61). Excess placental soluble fms-like tyrosine kinase 1 (sFlt1) may contribute to endothelial dysfunction, hypertension, and proteinuria in preeclampsia. Additionally, IL-1β recruits CD8^+^ T cells and neutrophils, causing damage to the villi (57). This recruitment also stimulates prostaglandin synthesis, which can lead to preterm labor (62).

Once IL-1β presses the “start button” for inflammation, TNF-α immediately takes the stage, acting as an “accelerator” that amplifies and sustains this inflammatory storm.

### 5.2 Tumor necrosis factor-alpha

Tumor necrosis factor-alpha (TNF-α) is a multifunctional pro-inflammatory cytokine that plays a role in immune regulation, apoptosis, and vascular function during both physiological and pathological processes in the placenta (63). In patients with chronic hypoxia-induced (including CHI) conditions, the TNF-α levels in placental tissue show a significant positive correlation with the risk of fetal growth restriction and preterm birth (64). TNF-α is primarily secreted by infiltrating macrophages and CD8^+^ T cells, with a smaller secreted by trophoblasts under stress conditions (65). In the placenta of cases with idiopathic FGR, TNF-α expression is significantly increased in trophoblastic giant cells and vascular endothelial cells. This increase may lead to fetal developmental restriction by inhibiting placental angiogenesis or directly damaging trophoblast function (66). In preeclampsia and gestational diabetes, elevated placental TNF-α levels is associated with insulin resistance, abnormal expression of advanced glycation end products and their receptor for advanced glycation end products. This pro-inflammatory microenvironment can induce localized oxidative stress and endothelial dysfunction in the placenta (67), which may lead to maternal hypertension and proteinuria (68).

TNF-α expression is essential for the formation of immune tolerance in early pregnancy. In the normal placenta development, TNF-α induces trophoblasts to express HLA-G, which inhibits the cytotoxicity of NK cells, thereby maintaining maternal-fetal immune tolerance (69). The soluble tumor necrosis factor receptor 1 (sTNFR1) secreted by placental tissue specifically neutralizes the pro-inflammatory effects of TNF-α, and this local immune regulation mechanism effectively suppresses autoimmune responses (70).

TNF-α-driven placental inflammatory signals can activate and amplify the complement system cascade reaction, which is a key marker of immune-mediated tissue damage.

### 5.3 The complement molecule C4d

C4d is a breakdown product activated by the classical complement pathway and is commonly associated with antibody-mediated immune responses (71). The deposition of the complement breakdown product C4d in placental inflammation is a significant pathological marker for pregnancy complications related to antiphospholipid antibodies (aPL) (72). Recent studies have shown that C4d plays a crucial role in organ transplant rejection and maternal-fetal immune tolerance (73). In the placenta of CHI, the deposition of C4d is distributed either diffusely or focally, and the amount of deposition is positively correlated with the severity of the disease (74). The abnormal expression of HLA class II molecules (such as HLA-DR and HLA-DQ) in placental trophoblast cells is significantly positively correlated with C4d deposition. This finding supports the idea that maternal anti-HLA antibodies may drive inflammatory responses and macrophage infiltration through the classical complement pathway (32).

The deposition of complement fragment C4d in CHI may be involved in the formation of pathological deposits in intercellular spaces due to the activation of the complement cascade (75). C4d is produced during the complement cascade and induces the formation of the membrane attack complex (MAC), which directly damages vascular endothelial and stromal cells while releasing anaphylatoxins C3a and C5a (76). Chemokines recruit monocytes, exacerbating local inflammatory responses. This leads to chronic inflammatory infiltration of the villous stroma and disruption of the epithelial barrier (77). Furthermore, C4d may enhance the pro-fibrotic microenvironment in conjunction with TGF-β. The deposition of C4d can directly promote the conversion of local fibrinogen to fibrin, exacerbating the formation of fibrotic networks in the villous space and the deposition of extracellular matrix, ultimately hindering maternal-fetal blood exchange (78).

In addition, although some studies have used C4d expression levels as an auxiliary diagnostic criterion, given lack of studies specifically investigating C4d as a diagnostic marker (27). the discriminatory value of C4d in CHI remains controversial (75). Along with C4d-mediated complement attack and inflammatory damage, changes in TGF-β signaling are also commonly observed in placental tissue. Changes in TGFβR1 expression and activity may reflect the body’s attempt to curb excessive inflammation and promote tissue repair.

### 5.4 Transforming growth factor beta receptor 1

In the placenta, transforming growth factor-β receptor 1 (TGF-βR1) acts as a receptor for transforming growth factor-β (TGF-β), playing a multifaceted role in regulating trophoblast differentiation, maintaining immune balance, and supporting angiogenesis to uphold pregnancy stability (79). TGF-βR1 and type II receptor (TβRII) are primarily expressed in the syncytiotrophoblast of the placenta, as well as in extravillous trophoblasts and the chorionic plate (80).

TGFβR1 is significantly upregulated in the placenta of CHI, and it may influence the pathological process of CHI by inhibiting trophoblast invasion, regulating maternal-fetal immune tolerance, and modulating inflammatory repair. It is a key cytokine that regulates the pathological changes in chronic placental inflammation (81). In preeclampsia, TGFβR1 is found to function in the remodeling of uterine spiral arteries by inhibiting trophoblast invasion (82). Besides, TGFβR1 plays an important role in immune tolerance and inflammatory repair at the maternal-fetal interface (83). In patients with recurrent miscarriage, the expression and activity of TGF-β are reduced, which can inhibit Treg proliferation and differentiation, leading to immune dysfunction at the maternal-fetal interface (84). The decreased ability of monocytes/macrophages to synthesize TGF-β1 fails to effectively suppress excessive inflammatory responses, leading to chronic inflammation (85). Furthermore, in the repair of inflammatory damage associated with chronic liver disease and pulmonary fibrosis, pro-inflammatory factors in the tissue microenvironment, such as TNF-α and IL-6, collaborate with TGF-β to induce fibroblast differentiation and extracellular matrix deposition (86).

### 5.5 Matrix metalloproteinases

Matrix metalloproteinases (MMPs) are a superfamily of proteases that depend on metal ions, such as zinc and calcium, as cofactors. They are capable of degrading critical components of the extracellular matrix (ECM), including collagen and elastin, leading to the disruption and remodeling of tissue structure, playing roles in placental development, immune regulation, and tissue remodeling (87). Tissue inhibitor of metalloproteinases (TIMPs) maintains tissue microenvironment homeostasis by inhibiting MMP-driven matrix degradation and excessive inflammatory responses, thereby preventing inflammation spread and tissue damage.

During tissue repair, MMP-2 participates in the clearance of damaged ECM, while TIMP-1 helps control the extent of degradation and promotes the deposition of new matrix (87, 88). Increased TIMP-1 activity is considered a key factor in promoting ECM accumulation and fibrosis formation (89). Abnormal MMP expression is the mechanism underlying the formation of massive perivillous fibrin deposition in CHI pathology (90–92). Additionally, TIMP-1 activation of the CD63/β1 integrin receptor complex in oligodendrocytes promotes the conversion of macrophages to an anti-inflammatory phenotype (M2 type), increasing IL-10 and TGF-β secretion while inhibiting the release of pro-inflammatory factors such as TNF-α and IL-6 (93).

In addition to MMPs, fibronectin is another important active molecule in the process of massive deposition around villi.

### 5.6 Fibronectin

Fibronectin deposition is a biomarker for diagnosing placental diseases and predicting recurrences (94). Fibronectin is a core molecule of the coagulation system, formed from fibrinogen after activation by thrombin. Its dynamic balance is crucial for hemostasis, inflammation, and tissue repair (95). In the placenta, fibronectin moderately deposits in the intervillous space, covering approximately 5%-10% of the area. This forms a temporary scaffold that supports the branching of the chorionic tree and the structural integrity of the maternal-fetal interface (96). Fibronectin promotes the fusion of trophoblast cells into a multinucleated syncytiotrophoblast through integrin (αvβ3) signaling, enhancing hormone secretion (such as hCG) and barrier function (97).

A significant feature of CHI pathology is the massive perivillous fibrin deposition (MPFD) around the chorionic villi. In this context, fibrin serves both as a product of inflammation-coagulation cross-reaction and as a key mediator driving placental damage (98). The maternal interface immune rejection activates mononuclear-macrophages, which release pro-inflammatory factors such as IL-6 and IL-1β. This process leads to endothelial damage in the intervillous space and thrombin generation, subsequently promoting the conversion and accumulation of fibrinogen into fibrin (99). Additionally, the necrosis of placental trophoblasts releases cellular debris and mitochondrial DNA. This activates Toll-like receptor 9 and the complement system, such as C5a, enhancing the local coagulation cascade and exacerbating fibrin deposition (100). Furthermore, under chronic hypoxic conditions, HIF-1α promotes the synthesis of plasminogen activator inhibitor-1 and inhibits plasmin activity. This ultimately hinders the clearance of fibrin (101). Together, these mechanisms contribute to increased fibrin deposition around the chorionic villi, negatively affecting placental function.

The impact of fibrin deposition covering the placental intervillous space on villous function manifests in two ways: physical obstruction and disruption of cellular signaling. The physical barrier created by fibrin mechanically hinders the exchange of oxygen and nutrients between mother and fetus. It also obstructs the migration of trophoblasts and immune cells, thereby suppressing placental development and villous vascularization (102). In cases of chronic hypoxia-induced (CHI), the area of fibrin deposition is negatively correlated with the birth weight percentile of newborns, indicating that fibrin accumulation affects placental exchange efficiency (103). Pathological observations reveal that in severe cases of CHI, the area affected by fibrin can reach 40%-60% of the placental volume, leading to extensive placental infarction and functional impairment (104). Furthermore, fibrin recruit macrophages and stimulates the activation of complement components C3d and C4d in the intervillous space, exacerbating immune rejection and inflammatory responses at the maternal-fetal interface (1).

## 6 Clinical assessment and research updates on CHI

### 6.1 Pathological diagnosis of CHI

CHI pathological diagnosis is based on placental histological examination, with routine examination content including observation of placental tissue morphology, CD68+ histiocyte positivity screening, and examination of fibrin deposition around the villi. Quantitative immunohistochemical analysis of placental pathology in CHI showed that the density of CD68+ macrophages in the CHI group was 88 ± 23 per unit area (HPF 40×), while in the control group it was 8 ± 5 per unit area (P < 0.001) (45). CHI pathology is classified into three grades—Grade 1 (5%-10%), Grade 2 (10%-50%), and Grade 3 (>50%)—which exhibit a significant dose-response relationship with perinatal outcomes (P<0.0001). The neonatal survival rate for Grade 3 patients was only 16.1%, significantly lower than that for Grade 2 (59%) and Grade 1 (86.5%) (P = 0.0002) (45). The risk of disease recurrence for Grade 2 and Grade 3 patients was 3.8 times higher than that for Grade 1 patients. Pathological grading is of great significance for predicting pregnancy outcomes and recurrence (105).

### 6.2 Biochemical diagnostics placental alkaline phosphatase

Placental alkaline phosphatase (PLAP) is an enzyme specifically expressed in placental cells. It regulates active transport across cell membranes and calcium-phosphate metabolism, providing nutrition to the fetus. There is academic debate regarding the clinical significance of alkaline phosphatase (ALP) levels in CHI patients from different regions. A retrospective cohort study from Canada involving 33 patients found that 31.6% (10/33) of CHI cases exhibited elevated serum ALP levels (>125 U/L), suggesting that ALP could serve as a reference indicator for inflammatory activity (106). However, a prospective cohort study from Japan in 2017 (n=58) conducted a multivariable regression analysis and found no significant correlation between fluctuations in ALP levels related to CHI (elevated group vs. normal group) and adverse pregnancy outcomes such as fetal growth restriction (OR = 1.32, 95% CI 0.75-2.34) or preterm birth (OR = 1.15, 95% CI 0.82-1.61) (p>0.05). This study also emphasized that the clinical value of PLAP as a specific diagnostic marker for CHI needs to be validated through large-scale multicenter studies (107).

### 6.3 HLA antibodies

The role of HLA antibodies as biochemical markers for CHI remains contentious within the international academic community. Some studies report that anti-paternal HLA-I/II antibodies may be detected in the placental tissue of CHI patients, with positivity rates reaching up to 75% in certain case reports (40). HLA antibodies may be associated with fluctuations in the expression levels of HLA-B and HLA-DRB1 alleles in peripheral blood. Additionally, some cases exhibit abnormal deposition of complement C4d in placental tissue (107). However, a recent cohort study from the UK found no statistically significant difference in the overall positivity rates of anti-HLA antibodies between the CHI group and healthy controls, indicating that the diagnostic specificity of a single HLA antibody marker has not yet met clinical requirements.

## 7 Progress in the treatment of CHI

Currently, there is no standardized treatment protocol for chronic histiocytic villous interstitial inflammation (CHI). However, several international clinical studies have shown that anticoagulation, anti-inflammatory, and biological therapies may have positive implications for improving pregnancy outcomes. Nevertheless, due to the limited sample sizes of existing studies and the lack of high-level evidence-based medical evidence, there remains significant controversy regarding the efficacy, safety, and applicability of various treatment methods.

Regarding anticoagulant therapy, research findings remain inconsistent (**Table 2**). A French clinical study involving 21 CHI patients showed that while the use of aspirin and low-molecular-weight heparin (LMWH) alone did not reduce the risk of preterm birth (still at 30%), the live birth rate significantly increased from 32% to 67% (12). In 2017, Japanese scholars proposed a triple therapy combining low-dose aspirin, corticosteroids, and LMWH. This strategy further improved the live birth rate and demonstrated superior clinical benefits compared to single anticoagulant regimens (107). A UK study involving 28 patients with refractory CHI demonstrated that adding hydroxychloroquine (200 mg/day) and prednisolone (20 mg/day) to aspirin (75–150 mg/day) and LMWH therapy significantly improved the live birth rate (from 61.5% to 86.2%, p < 0.05) (11). However, due to the small sample sizes of all studies and the lack of randomized controlled trials, the exact efficacy and applicability of combined anticoagulation and immunosuppression therapy in a broader population remain controversial.

**Table 2:** Treatment strategies for CHI.

Treatment category	Representative regimen	Mechanism of action	Reported efficacy
Anticoagulation	Aspirin + LMWH	Antithrombotic, improves placental perfusion	Improved live birth rate (32%→67%) (5)
Anti-inflammatory	Aspirin + LMWH + Corticosteroids	Anticoagulation + Immunomodulation	Improved live birth rate, superior combined effect (102)
	Aspirin + LMWH+ Hydroxychloroquine + Prednisolone	Anticoagulation, Immunomodulation, Anti-inflammation	Live birth rate 86.2% (vs 61.5%) (4)
Biological Agents	Adalimumab (anti-TNF-α)	Blocks inflammatory signaling pathway	Clinical response rate 72.3% (103)
	Anakinra + Colchicine	Inhibits NLRP3 inflammasome	Improved perinatal outcomes (54)

CHI, Chronic Histiocytic Intervillositis; LMWH, Low Molecular Weight Heparin; TNF-α, Tumor Necrosis Factor-alpha; IL-1, Interleukin-1.

In terms of anti-inflammatory therapy, immune modulation strategies are primarily used to suppress excessive inflammatory responses. Glucocorticoids are commonly used drugs and are often combined with anticoagulants. Studies in Japan and the UK have suggested that adding glucocorticoids can further improve live birth rates and expand clinical benefits. Hydroxychloroquine is also commonly used to inhibit immune inflammatory pathways, and its combination with low-molecular-weight heparin and glucocorticoids has shown synergistic anti-inflammatory and immunomodulatory effects (11). However, this class of treatment still faces challenges such as significant individual response variability, unclear long-term safety, and lack of consensus on optimal treatment regimens.

In terms of biological therapy, agents targeting specific inflammatory factors or signaling pathways offer new directions for refractory CHI. Anti-TNF-α monoclonal antibodies such as adalimumab (40 mg every two weeks) achieve a clinical remission rate of 72.3% in refractory cases, with the mechanism involving blocking the TNF-α signaling pathway, inhibiting abnormal macrophage activation, and reducing inflammatory infiltration in the villous spaces (108). The IL-1 receptor antagonist anakinra combined with colchicine can inhibit NLRP3 inflammasome activation, and reports indicate it can improve perinatal outcomes in patients with recurrent CHI (59). However, such biologics are currently limited to case reports or small case series, and their safety, timing of administration, and long-term maternal and infant outcomes require further research validation.

Additionally, various natural immune modulators exhibit good anti-inflammatory effects and have the potential to become drugs for CHI treatment. The flavonoid quercetin effectively improves endothelial dysfunction in preeclampsia (109, 110).the flavonoid hesperidin exhibits excellent primary villus antioxidant activity (111). Research indicates that liposoluble vitamin D3 is a key regulatory factor in placental and fetal development (112, 113). Additionally, plant estrogens such as soy isoflavones can inhibit Th17 expression in the placenta, promote Treg expansion, reduce CD68^+^ macrophages in the placenta, and mitigate inflammatory responses (114).

## 8 Discussion

**Figure 1:**
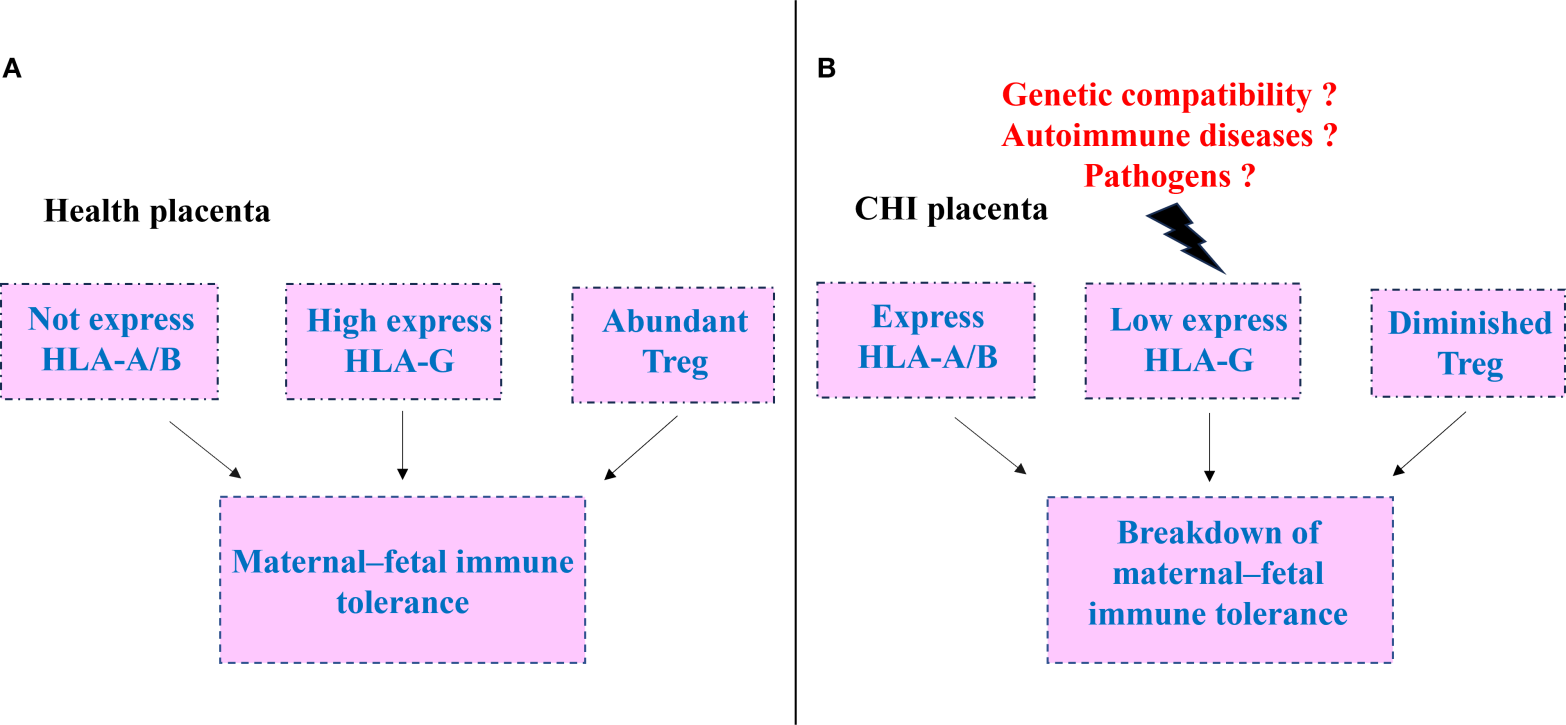
Schematic of tolerance in normal pregnancy and its breakdown in chronic histiocytic intervillositis (CHI). **(A)** Normal maternal -fetal immune. extravillous trophoblasts express high levels of HLA-G, lack HLA-A/B expression, and are accompanied by abundant regulatory T (Treg) cells, sustaining immune tolerance at the maternal -fetal interface. **(B)** Tolerance collapse in CHI.HLA-G expression is markedly diminished, HLA-A/B is up-regulated, and Treg cells are reduced, precipitating the breakdown of maternal -fetal immune tolerance.

Chronic histiocytic intervillositis (CHI) is a rare, placenta-specific immune-inflammatory disorder characterized by disrupted maternal–fetal immune tolerance. The maintenance and breakdown of immune tolerance at the maternal–fetal interface are regulated by multiple factors, including genetic compatibility, maternal autoimmune status, and infections (**Figure 1**). During the induction of immune tolerance in normal pregnancy, HLA molecules play an important role. Trophoblasts do express classical HLA-A/B molecules but highly express non-classical HLA-G, which acts in coordination with locally enriched regulatory T cells (Tregs) to effectively suppress maternal immune activation and maintain immune tolerance. Under conditions such as poor histocompatibility between mother and fetus, autoimmune diseases, or pathogen infection, the tolerant balance at the maternal–fetal interface is disrupted. Trophoblasts aberrantly upregulate HLA-A/B expression, exhibit reduced HLA-G levels, and are accompanied by a decrease in Treg numbers, collectively leading to a breakdown in maternal–fetal immune tolerance and a shift toward pathological responses as CHI.

**Figure 2:**
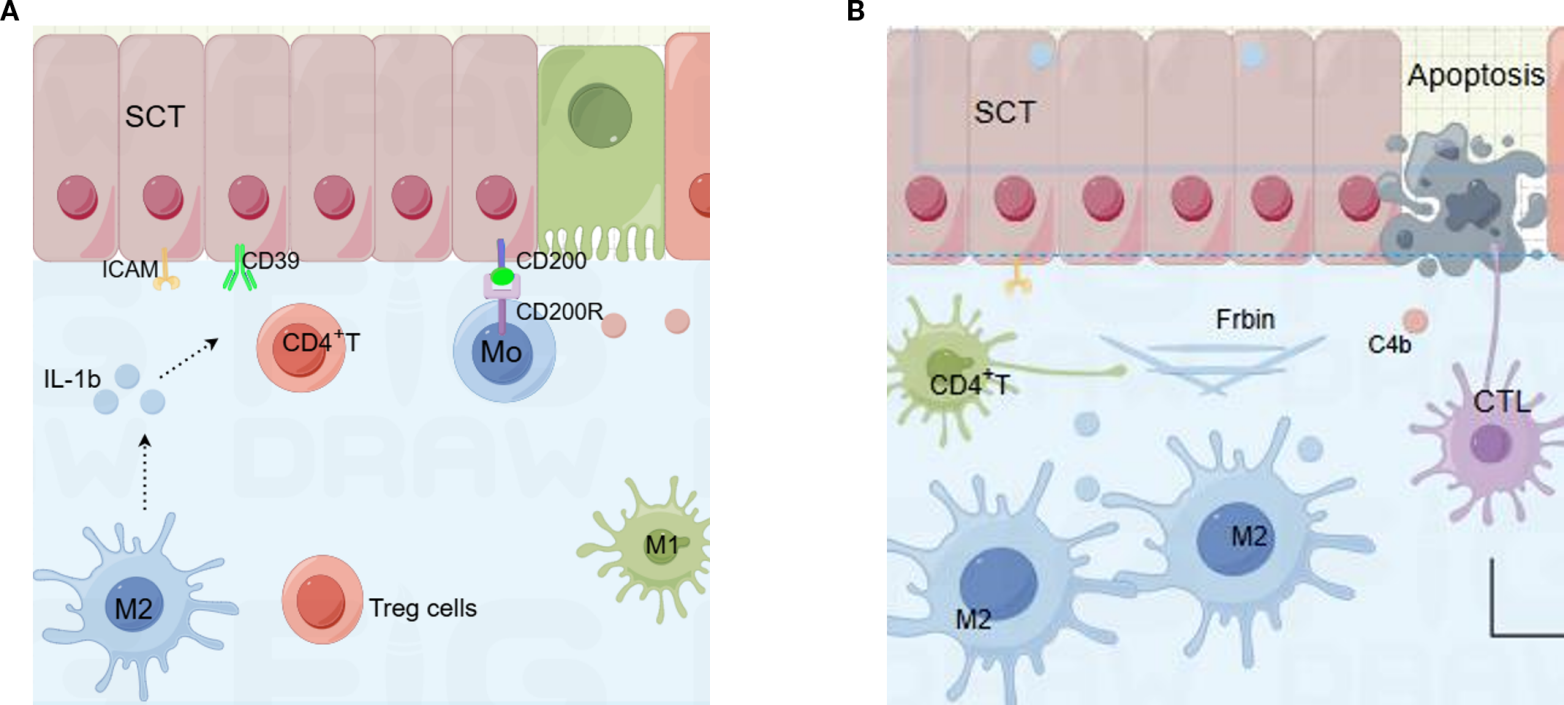
Cellular and molecular components in two processes of chronic histiocytic intervillositis (CHI). **(A)** Initiators of inflammation in CHI. Cells includes the syncytiotrophoblast (SCT), macrophages, CD4⁺ T cells, and soluble meditheators including interleukin-1β (IL-1β), CD200 -CD200R axis, ectonucleoside triphosphate diphosphohydrolase-1 (CD39), and intercellular adhesion molecule-1 (ICAM-1). **(B)** Mediators of injury and repair in CHI: fibrin deposition and complement component 4b (C4b). SCT, syncytiotrophoblast; IL-1β, interleukin-1β; ICAM-1, intercellular adhesion molecule-1; C4b, complement component 4b.

CHI represents an aberrant maternal immune response to semi-allogeneic fetal antigens. This review summarizes the cellular and molecular players implicated in the pathogenesis of CHI and proposes a two-phase model (**Figure 2**): (A)inflammation initiation and (B)tissue repair. During the initiation phase, down-regulation of CD200 and CD39 on syncytiotrophoblasts compromises maternal–fetal tolerance. Concomitantly, interleukin-1β (IL-1β) is up-regulated and secreted into the interstitial space, triggering an inflammatory cascade. Macrophages infiltrate the placental bed and polarize, while T cells migrate and become activated. In the subsequent repair phase, monocytes differentiate into M2 macrophages. Chronic inflammation promotes the release of cytokines such as gasdermin D, inducing tissue cell apoptosis. Extensive deposition of complement split product C4d and fibrin results in abundant perivillous fibrinoid material.

## 9 Outlook

Looking ahead, research on the mechanisms, diagnosis, and treatment of CHI is gaining momentum, with several key areas for future investigation outlined below:

Some immunosuppressants have shown promising therapeutic effects. There is a good application potential for developing inhibitors targeting key proteins involved in CHI regulation, such as chemokines and NLRP3 inflammasome pathways.

The serological diagnosis of CHI aims to develop non-invasive biomarkers based on cell-free fetal DNA or exosomes from maternal blood, including HLA antibodies and specific microRNAs.

Some immunosuppressants have shown promising therapeutic effects. There is a good potential for developing inhibitors targeting key proteins involved in CHI regulation, such as chemokines and the NLRP3 inflammasome. Additionally, we will explore the use of placenta-derived mesenchymal stem cells or gene-edited CAR-Treg cells for localized delivery to the chorionic space to promote immune tolerance.
